# Are income-related differences in active travel associated with physical environmental characteristics? A multi-level ecological approach

**DOI:** 10.1186/s12966-015-0217-1

**Published:** 2015-06-05

**Authors:** Esther Rind, Niamh Shortt, Richard Mitchell, Elizabeth A Richardson, Jamie Pearce

**Affiliations:** Centre for Research on Environment, Society and Health (CRESH), Institute of Geography, School of GeoSciences, University of Edinburgh, Edinburgh, UK; Centre for Research on Environment, Society and Health (CRESH), Institute of Health and Wellbeing, University of Glasgow, Glasgow, UK

**Keywords:** Active travel, Urban areas, Physical environment, Health inequality, Ecological analysis

## Abstract

**Background:**

Rates of active travel vary by socio-economic position, with higher rates generally observed among less affluent populations. Aspects of both social and built environments have been shown to affect active travel, but little research has explored the influence of physical environmental characteristics, and less has examined whether physical environment affects socio-economic inequality in active travel. This study explored income-related differences in active travel in relation to multiple physical environmental characteristics including air pollution, climate and levels of green space, in urban areas across England. We hypothesised that any gradient in the relationship between income and active travel would be least pronounced in the least physically environmentally-deprived areas where higher income populations may be more likely to choose active transport as a means of travel.

**Methods:**

Adults aged 16+ living in urban areas (n = 20,146) were selected from the 2002 and 2003 waves of the UK National Travel Survey. The mode of all short non-recreational trips undertaken by the sample was identified (n = 205,673). Three-level binary logistic regression models were used to explore how associations between the trip being active (by bike/walking) and three income groups, varied by level of multiple physical environmental deprivation.

**Results:**

Likelihood of making an active trip among the lowest income group appeared unaffected by physical environmental deprivation; 15.4% of their non-recreational trips were active in both the least and most environmentally-deprived areas. The income-related gradient in making active trips remained steep in the least environmentally-deprived areas because those in the highest income groups were markedly less likely to choose active travel when physical environment was ‘good’, compared to those on the lowest incomes (OR = 0.44, 95% CI = 0.22 to 0.89).

**Conclusions:**

The socio-economic gradient in active travel seems independent of physical environmental characteristics. Whilst more affluent populations enjoy advantages on some health outcomes, they will still benefit from increasing their levels of physical activity through active travel. Benefits of active travel to the whole community would include reduced vehicle emissions, reduced carbon consumption, the preservation or enhancement of infrastructure and the presentation of a ‘normalised’ behaviour.

## Background

There is growing evidence that active travel (walking or cycling for non-recreational purposes, including trips undertaken for commuting, business, shopping etc.) can contribute significantly to levels of overall physical activity [1, 2], with associated benefits for health [3]. Even short bouts of activity have been shown to contribute to physical and mental well-being [3, 4]. Yet, the average annual distance actively travelled per person in the UK actually decreased by 28 % (from 306 to 221 miles) since the 1970s [5] (though this decline may be slowing or even reversing [6]). For many local journeys, walking or cycling could be a reasonable alternative to motorised transport and, in addition to conveying health benefits to the participant, would contribute to a reduction in traffic-related air pollution, accidents, and carbon use, as well as helping to normalise physical activity [2, 7]. These environmental and health-related co-benefits emphasise the potential importance of creating an environment conducive to active transport [7].

The environmental and social determinants of health behaviours, including active travel, have been conceptualised using ecological models [8, 9]. These comprise ‘layers’ of influence including individual biological (e.g. age), psychological (e.g. attitudes towards physical activity), intrapersonal (e.g. social support) influences, and broader social and physical environmental factors [10]. Prior research has explored aspects of the social environment, such as levels of social capital and perceived safety of an area [10, 11] but research on the physical environment has been largely focused on examining built environment features, including functional patterns (e.g. street connectivity), safety issues (e.g. heavy traffic), aesthetic components (e.g. maintenance of green spaces), and destination accessibility (e.g. proximity to shops) [12]. Less is known about how active travel patterns may be related to ‘natural’ physical environmental factors such as air pollution, climate and green space.

A small study of residents from the San Francisco Bay area revealed that exogenous factors such as topography, darkness and rainfall had stronger associations with walking and cycling than did established characteristics of the built environment including street connectivity, land use mix, and proximity to retail facilities [13]. Other results from the US indicate that active transport is higher in areas with access to national parks, forests and blue space, and that greater participation in cycling is associated with aspects of moderate climate, topography, and low levels of air pollution [14]. The latter has been a concern to on-road cyclists, a group more likely to be exposed to harmful levels of air pollutants [15] potentially increasing the risk of respiratory and cardiovascular morbidity and mortality [16]. Physical features including pleasant views of gardens, roadside greenery and other green spaces, as well as low air pollution, may also encourage older people to walk to destinations such as shops or community services [17].

Some research has also reported a socio-economic gradient in active travel, with higher levels often found in the most socio-economically deprived groups [18]. However, there is a less than consistent relationship between active travel and socio-economic position; it appears to vary by place and time [19–21]. Where higher levels of active travel are found among more deprived populations, it has been attributed to a lack of material resources including car access, resulting in greater dependency on modes of active travel [18, 22]. Although there have been calls for a more integrated analysis of all determinants of active transport [23], including the social and the physical environment [24, 25], little is known about whether, and how, the income-related social gradient in active travel patterns may be related to physical environment. A better understanding might aid the development of tailored interventions aiming to increase levels of physical activity in particular population subgroups, by considering their socio-economic circumstances as well as characteristics of their local environment.

In previous research we explored the association between physical activity and multiple aspects of the physical environment using an index of multiple environmental deprivation (MEDIx) [26] derived for the year 2001. The index consisted of aspects of the environment that are both health damaging (air pollution, proximity to industry and cold climate) and health enabling (green space and UV levels) [27]. Results demonstrated respondents were most likely to engage in active travel, and specifically walking for transport, in the most physically-deprived environments. The ‘choice’ to engage in active travel is not made in isolation, but rather reflects broader socio-ecological environments, alongside individual characteristics. For the most economically deprived populations, the affordability of mechanised transport may constrain an individual’s choice and as such, render levels of active travel within this population group unrelated to broader physical environment. Yet, a more conducive physical environment might encourage those less dependent on active transport to choose walking or cycling over mechanised transport. If this hypothesis is correct, we would expect the social gradient in active transport to be reduced in areas with better physical environments. It is likely that the most deprived populations will still have to actively travel, but a better quality environment may encourage more affluent groups to actively travel, and hence reduce the inequality. The relationship between multiple aspects of physical environment and socio-economic inequalities in active travel remains unexplored.

To address this knowledge gap, we explored income-related differences in active travel in relation to physical environmental disadvantage in urban areas across England. We expected the income-related gradient in active travel to be less pronounced in the least environmentally-deprived areas where, in comparison with more environmentally-deprived areas, more affluent individuals may be more likely to choose active travel.

## Methods

### Survey data

Active travel data were taken from the National Travel Survey (NTS), a nationally-representative cross-sectional survey first commissioned by the Ministry of Transport in 1965 to monitor long-term changes in individual travel patterns in Great Britain [28]. The full sampling and interview methodology are described elsewhere [29]. In short, face-to-face interviewing was used to collect key socio-economic, demographic and travel-related characteristics of participants. A subgroup of individuals completed a travel diary recording trips undertaken over the course of a week. To boost the sample size for statistical analysis, we pooled data from the survey waves 2002 and 2003 (n = 42,817, including 33,717 adults aged 16+ and 9,100 children <16 years) which matched the timeframe of the available environmental data. Whilst acknowledging the age of these data, these survey years were selected to closely match the time period for which the measure of physical environment was available. Our final sample included all participants of the diary sub-sample (age 16+) with full information on active travel, living in urban areas (n = 20,146).We opted to include those aged 16 and 17 in our definition of adults as they are potentially working people, and are normally making independent journeys and travel decisions at these ages. Those aged over 17 are also eligible to drive in the UK. Note that ethical approval was not required for the analysis of this publicly available, anonymised secondary dataset.

#### Active travel

We defined active travel as walking or cycling for non-recreational purposes, including for commuting, business, education, shopping and any other personal business. We measured such active travel at ‘trip’ level. In this case a trip is defined as a one-way course of travel from one place to another with a single main purpose. First we identified all trips (active and mechanised) with a non-recreational purpose. We then selected trips for which there might reasonably be a ‘choice’ over mode: we excluded trips that were so short as to be almost certainly walked or biked, defined as less than 1/10th of a mile (160 m), and trips that were so long that active travel was likely only by cycling enthusiasts, defined as more than 5 miles (8 km). These thresholds were defined based on the distribution of active travel mode within the data; almost all trips less than 1/10th mile (160 m) were active, but almost none more than 5 miles (8 km) were active. We considered separate analysis for cycling only, but numbers were too low. This approach identified 205,673 trips of interest. The resulting binary outcome variable then assessed whether a trip of interest was made actively (i.e. by walking or cycling) or not (i.e. by mechanised means). The mode of travel referred to the main mode, i.e. that which was used to complete most of the journey.

#### Trip-, Individual-, household- and area-level covariates

We selected a range of covariates known to be associated with active travel. All models were adjusted for trip distance (in miles), age group (16 to 29, 30 to 49, 50 to 69, and 70+), sex (male/female), ethnicity (White/non-White), self-reported walking difficulties (yes/no), car access (yes/no), bicycle access (yes/no) and household income (<£25,000, £25,000 to £49,999, and £50,000+). Previous research has shown that residents living in socio-economically deprived neighbourhoods were more likely to actively travel than their less deprived counterparts [18]. Hence, we included the Carstairs Deprivation Index 2001, a well-established and robust area-level measure of socioeconomic deprivation including low social class, lack of car ownership, overcrowding and male unemployment [30]. We were concerned about the inclusion of car ownership in the area-level measure of deprivation since it is known to be skewed by urbanity and may also reflect alternative transport options (active or public transport) in the neighbourhood. However, there was no other measure available for the whole UK, on a consistent basis for this time period. Although our sample was urban residents only, we included a measure of urban settlement size to adjust our analysis for increased public transport demand and provision in more densely populated urban areas [31,32]. Based on their area of residence, each household of the survey was assigned to an urban category including very large (population >250,000), large (population >10,000 to 250,000) and smaller (population over 3,000 to 10,000) urban areas [29]. Other correlates, plausibly associated with active travel, were also explored. These included interview season, employment status, and having children <16 years in the household. These were not significantly associated with active travel (p > 0.05) and were thus excluded from further analysis.

The appropriate weights for trip-level analyses were provided by the NTS and were applied to all models. These accounted both for the drop-off in the number of trips recorded by participants over the course of the week and non-response of households to the survey [29].

#### Measuring environmental deprivation

Physical environment was measured using the multiple environmental deprivation index (MEDIx) for UK Census Area Statistic (CAS) wards (n = 10,654). The development of this indicator is described elsewhere [27, 32]. In short, MEDIx assesses environmental dimensions both detrimental (air pollution, proximity to industry and cold climate measures) and beneficial (green space availability and UVB measures) to health. MEDIx scores range from −2 (least deprived) to +3 (most deprived). Due to small numbers at the extreme ends of the MEDIx scale, we combined categories MEDIx +2 and +3 into one group, and MEDIx categories −1 and -2 into another, leaving four categories of environmental deprivation. For each of the NTS respondents a CAS ward of residence identifier was obtained which made it possible to link information on individual active travel patterns to physical environmental deprivation. For reasons of confidentiality, survey respondents living in wards with an individually-identifiable combination of environmental characteristics were excluded from this study [n = 4,538 (18.4%)]. In comparison to the original dataset, the final dataset (n = 20,146) comprised more adults (+4.1%) living in very large urban areas with a population of >250,000, but fewer observations (−2.4%) in urban areas with a population of >10,000 to 250,000. All other socio-demographic differences between the included and excluded participants were ≤ ±2.0%.

Finally, we also explored adjusting for region of residence, and in particular for residence in London given its unique urban structure and public transport infrastructure. Analyses showed, however, that i) relationships between active trips and income did not seem to vary significantly by residence in Greater London but also ii) that there were collinearities between this variable and MEDIx score, Carstairs score and urban category. We therefore opted not to adjust for region.

### Statistical analysis

#### Descriptive statistics

First, we tested univariate associations between active trips and all covariates using *χ*^2^-tests. We then explored the relationship between income and active trips across different categories of environmental deprivation using a graph.

#### Multivariate, multi-level models

We ran a three-level binomial logistic random-intercept model, predicting the choice of active mode for a trip, where trips (level 1) were nested within individuals (level 2, nested in CAS wards (level 3). The trip weight included a household weight which allowed us to run a three-level model. Models were run first without covariates and were then adjusted. We initially stratified the analysis by MEDIx category to explore the nature of any interactions, following an approach previously applied in health-related research e.g. [33,34]. A full non-stratified model was then run, including interaction terms for income group and MEDIx to establish formally whether income-related differences in active trip mode varied significantly (at a significance level of p < 0.05) by physical environmental deprivation. The lowest income group (less than £25,000) and environments with an intermediate level of environmental deprivation (MEDIx 0) were used as reference categories. This choice was based on associations between household income, active trips and environment observed at the descriptive statistics stage. We checked the impact of our reference category choices by repeating analyses using alternatives, but there were no substantive differences. All statistical analyses were run in Stata/IC 12.1 [35].

## Results

### Characteristics of the sample in relation to active trips

About 13% of the 205,673 trips were active (Table 1). Further characteristics of the study sample and univariate associations with trip mode are summarised in Table 1.

**Table 1:** **Characteristics of the study sample in relation to making a trip of interest (0.1 to 5 miles) by active means, including adults aged 16+ from urban areas, National Travel Survey 2002 and 2003**

	**All trips (active & motorised)**		**Active trips (cycling or walking)**			
	^**†**^ **n**_**weighted**_	**%**	^**†**^ **n**_**weighted**_	**% active**	**Pearson chi** ^**2**^	***p*** **-value**
**Age group**					2600.0	<0.001
16-29	55,047	26.8	6,166	11.2		
30 - 49	88,960	43.3	10,120	11.4		
50 - 69	41,651	20.3	8,985	21.6		
70+	20,015	9.7	2,382	11.9		
Total	205,673	100.0	27,652	13.4		
**Sex**					119.7	<0.001
Males	93,521	45.5	13,618	14.6		
Females	112,152	54.5	14,034	12.5		
Total	205,673	100.0	27,652	13.4		
**Ethnicity**					38.4	<0.001
White	189,176	92.0	25,239	13.3		
Non-White	16,486	8.0	2,408	14.6		
Total	205,662	100.0	27,647	13.4		
Missing	11	<0.1%	5			
**Walking difficulties**					371.7	<0.001
Yes	22,148	10.8	2,015	7.9		
No	183,505	89.2	25,633	14.0		
Total	205,654	100.0	27,648	13.4		
Missing	19	<0.1%	4			
**Car access**					8800.0	<0.001
Yes	175,691	85.4	18,736	10.7		
No	29,982	14.6	8,916	29.7		
Total	205,673	100.0	27,652	13.4		
**Bike access**					1100.0	<0.001
Yes	80,576	39.2	14,224	17.7		
No	125,088	60.8	13,424	10.7		
Total	205,665	100.0	27,648	13.4		
Missing	8	<0.1%	4			
**Journey distance**					21000.0	<0.001
<1 mile	20,291	9.9	1,047	5.2		
1 to < 2 miles	72,991	35.5	20,392	27.9		
2 to < 3 miles	50,474	24.5	4,208	8.3		
3 to < 5 miles	61,918	30.1	2,006	3.2		
Total	205,673	100.0	27,652	13.4		
**Household income**					849.9	<0.001
less than £25,000	99,289	48.3	15,603	15.7		
£25,000 to £49,999	74,631	36.3	8,802	11.8		
£50,000 and over	31,753	15.4	3,248	10.2		
Total	205,673	100.0	27,652	13.4		
**Socio-economic deprivation**					783.5	<0.001
(Carstairs tertiles)						
1 (least deprived)	67,033	32.6	6,978	10.4		
2	70,578	34.3	9,887	14.0		
3 (most deprived)	68,062	33.1	10,788	15.8		
Total	205,673	100.0	27,652	13.4		
**Urban classification**					221.3	<0.001
population over 3 k to 10 k	10,740	5.2	1,323	12.3		
population over 10 k to 250 k	88,373	43.0	12,876	14.6		
population over 250 k	106,561	51.8	13,453	12.6		
Total	205,673	100.0	27,652	13.4		
**Environmental deprivation**					93.6	<0.001
(MEDIx* category)						
−2/-1 (least deprived)	19,441	9.5	2,590	13.3		
0	53,786	26.2	7,755	14.4		
+1	101,770	49.5	13,554	13.3		
+2/+3 (most deprived)	30,676	14.9	3,753	12.2		
Total	205,673	100.0	27,652	13.4		

†Results weighted for sample and trip bias (see Methods).

*Multiple Environmental Deprivation Index, capturing small-area exposure to multiple health-related environmental characteristics including air pollution, proximity to industry, cold climate, green space and UVB.

**Figure 1:**
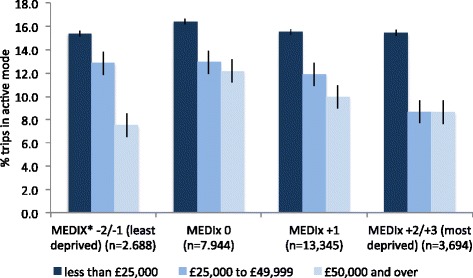
Percentage of trips of interest (0.1 to 5 miles) made by active means, stratified by income group and environmental deprivation. *Multiple Environmental Deprivation Index, capturing small-area exposure to multiple health-related environmental characteristics including air pollution, proximity to industry, cold climate, green space and UVB.

Whilst all of the bivariate associations were significant, this is likely to have been a consequence of the large sample size. Unsurprisingly, not having walking problems, having a bike, and not having a car appeared strongly related to making an active trip. Trip distance was also strongly negatively associated with active mode. Of particular interest for this analysis was the negative association between household income group and active trip choice. However, associations between MEDIx and active trips were more modest, with no clear gradient.

(Figure 1) presents the relationship between active trip mode and income across the categories of environmental deprivation. These are unadjusted values, but in fact give a very clear picture of the results obtained from the adjusted models. An income-related gradient in active trips was clear across all of the MEDIx categories, with the lowest income group always reporting the highest levels of active trips. Figure 1 suggests that choosing active trip mode was barely affected by environmental deprivation for those in the lowest income group. Indeed, the proportion of non-recreational trips being made actively in the least and most environmentally deprived areas was the same, at 15.4 %. However, Figure 1 also suggests that the middle and higher income groups did show some sensitivity to environmental deprivation. The middle income group was increasingly less likely to choose an active mode for their trips as environmental deprivation worsened. The high income group followed this pattern too, but only as MEDIx score worsened from 0 to +2/+3. In the least environmentally deprived areas (MEDIx −2/-1) the high income group showed a marked and significantly *lower* likelihood to choose an active mode. Table 2 presents results from stratified models which confirm that adjustment for covariates did not alter the observed associations. Compared to the lowest income group, the odds of choosing to make an active trip reduced with increasing environmental deprivation among those in the middle income group. Yet, they were also significantly lower among the highest income group living in the *least* environmentally deprived areas (OR = 0.44, 95% CI = 0.22 to 0.89). Not surprisingly, the full model showed no evidence of a significant interaction in the association between household income and active trips, by environmental deprivation (results not shown).

**Table 2:** **Odds ratios for the likelihood of trips of interest (0.1 to 5 miles) being made by active means in the middle and highest income groups, relative to the lowest income group, stratified by environmental deprivation**

	**Groups of environmental deprivation**														
	**MEDIx^ -2/-1**			**MEDIx 0**				**MEDIx +1**				**MEDIx +2/+3**			
	**(least deprived)**											**(most deprived)**			
	**OR**	**95% CI**		**OR**		**95% CI**		**OR**		**95% CI**		**OR**		**95% CI**	
**Unadjusted models**															
Less than £25,000	1.00			1.00				1.00				1.00			
£25,000 to £49,999	**0.60**	0.45	0.81	*0.79*		0.63	0.98	**0.68**		0.58	0.81	**0.53**		0.39	0.71
£50,000 and over	**0.32**	0.18	0.56	*0.66*		0.48	0.90	**0.51**		0.40	0.64	**0.43**		0.29	0.62
**Adjusted for individual variables & contextual covariates**															
Less than £25,000	1.00				1.00				1.00				1.00		
£25,000 to £49,999	0.97	0.60	1.56		0.92	0.73	1.17		*0.81*	0.67	0.99		*0.68*	0.48	0.95
£50,000 and over	*0.44*	0.22	0.89		0.87	0.62	1.21		**0.62**	0.48	0.81		*0.57*	0.36	0.89

Bold = significant at p < 0.01; *italics =* significant at p < 0.05.

^Multiple Environmental Deprivation Index, capturing small-area exposure to multiple health-related environmental characteristics including air pollution, proximity to industry, cold climate, green space and UVB.

## Discussion

Regardless of levels of physical environmental deprivation, those in the lowest income groups had greater odds of making active non-recreational trips. Contrary to our expectations however, the income-related gradient in making active trips remained as steep in the least environmentally-deprived areas as in the most environmentally-deprived areas. This reflects the fact that those in the highest income groups were markedly less likely to choose active travel when the physical environment was good. Within the limits of a cross-sectional study design, the results suggest that improving physical environment is unlikely to mitigate socio-economic differences in active travel.

To our knowledge this was the first study assessing variation in income-related inequalities in active travel by *multiple* characteristics of the physical environment. The particular advantage of the MEDIx score is that it enables us to look not only at negative aspects of physical environment, such as air pollution and cold climate, but positive aspects such as green space. Our results suggest that where environment is adverse, those whose incomes may enable them to choose *not* to actively travel, are indeed more likely to do so. Yet, the most benign or favourable physical environment does not seem to encourage active travel among the same higher income groups.

Previous research had suggested that neither the social [11] nor the built-environment [20] are major factors in determining active transport behaviours in socio-economically deprived populations. For example, in a study on active travel amongst a deprived urban population group in the UK, Ogilvie et al. [21] concluded that measures of the local environment did not explain much of the variance in active travel. Their research points to the fact that such population groups *‘may simply have adapted to adverse conditions in their local environment’* [21]. It may be that the higher levels of active travel among those on low incomes in our study were also evidence of ‘adapting’ to such adverse conditions. Such ‘adaptation’ raises questions of risk and harm. Literature on active travel has discussed whether the benefits of active travel always outweigh the associated costs and risks, in particular focussing on active travel in adverse environments. We know that risks of active transport include increased mortality and morbidity from traffic accidents [16], the mental drain of having to rely on such forms of transport in stressful environments [23] as well as exposure to air pollution [7] which is related to cardiovascular mortality and respiratory conditions [36]. Gatrell [37] recently highlighted that all modes of transport, including walking and cycling, have ‘dis-benefits’. Previous research has also shown that health is worse in areas with higher levels of multiple environmental deprivation [27]. It is pertinent to question whether higher levels of active travel in areas of extreme environmental deprivation are wholly health-enabling. However, whilst there may be risks associated with active travel, the estimated health benefits substantially outweigh the risks relative to car driving [16]. Nevertheless, policies aiming to increase active transport also need to consider the risks involved, particularly for the most income-deprived populations in the most environmentally-deprived areas.

The low levels of active travel among the affluent in good physical environments is intriguing and of concern. It may reflect the ubiquity of motorised transport among socio-economically advantaged groups [6]. It is also possible that the combination of higher income and MEDIx score −2/-1 is capturing residence in environments which are different somehow to those with the same MEDIx score but occupied by those on middle or lower incomes. Whilst everyone in the sample lived in an urban area, and we adjusted for size of settlement, these crude measures cannot capture the other environmental characteristics which may influence active travel such as walkability, or the nature and number of destinations.

Does a low level of active travel for the more advantaged really matter? Whilst the affluent are more likely to reach recommended levels of physical activity through recreational activity and to retain a whole host of other health advantages over more deprived populations, we believe this is an important issue for at least two reasons. First, we are not just interested in the health of those who do or do not participate in active travel. Population health reflects the social and physical environment that everyone shares and contributes to. We know that the benefits of active travel to the non-participating community include reduced vehicle emissions, reduced carbon consumption, the preservation or enhancement of infrastructure and the presentation of a ‘normalised’ behaviour. If one group does not participate in active travel, this affects the health of others. Second, physical activity may reduce the risk of health outcomes which are not strongly socially patterned; mental health and wellbeing for example. Thus, just because a more affluent group seems systematically less likely to participate in active travel and they may on some health measures, be relatively advantaged, this does not mean that their health could not or should not be improved. Public health has a duty to maximise population health, not just minimise inequalities. We suggest therefore, that policy options to reduce private transport must target the more affluent. The related co-benefits will be felt by the whole of the population through the reduction of broader environmental concerns.

Key strengths of this paper include its use of the nationally representative National Travel Survey (NTS), designed to measure travel behaviour, the use of a robust multiple environmental deprivation measure which captures both ‘good’ and ‘bad’ physical environments, a large sample size, adjustment for a good range of potential confounders as identified by the literature and the use of multilevel models to allow for sample design. Alongside these however, were several limitations. The data used in the paper were relatively old. This was necessary because whilst the NTS is a regularly repeated survey, the measure of physical environmental deprivation was only available in 2001. It was essential to measure the environment and behaviour at about the same time. It is plausible that both physical environments, and active travel behaviour have changed since this time period and indeed, recent work in the UK has suggested some minor increases in this behaviour, but that socio-economic differences remain stark [6]. Whilst more up-to-date data would be desirable, it does not appear that there has been a substantial shift either in the levels, or the socio-economic distribution, of active travel sufficient to warrant our results irrelevant.

Effect sizes did not change substantially with adjustment for individual- and area-level confounders. However, other factors not included in this study may be more important in explaining the relationship between household income and active travel. These could include individual psychological measures such as personal attitudes, perceptions, motivations and preferences related to active travel [24]. Furthermore, it is possible that these factors are patterned by individual-level socio-demographic covariates included in our analysis. The extent to which residual confounding remains in our results is thus not clear.

The study modelled trips in which the main form of travel was active, rather than the mode of travel on each leg of each trip. This approach was taken because of the computational complexity of trying to model all legs on 205,673 trips. However, we acknowledge that mixed-mode journeys, for example walking to public transport, then taking it, then walking from the terminal to the destination, will not have been well handled. This was a very large study and what we have lost in detail by modelling trips, is arguably offset by the gain in modelling at a population scale, and with a range of physical environments.

The area-based measure of socio-economic deprivation deployed was not ideal as it included car ownership levels. However, the Carstairs Index is one of the most widely used and tested measures in the UK and it is known to be effective at identifying socio-economic situations likely to affect health and related behaviours. Use of the Carstairs Index is highly unlikely to have affected our substantive findings.

Although our outcome measure was based on self-reported travel mode, which may make it vulnerable to over-reporting of active travel levels [38], the prevalence of active transport in our study sample was relatively low (~13%) , and was similar to comparable findings from the US [39]. There is a substantial literature comparing self-reported levels of physical activity with those measured by objective devices such as accelerometers, and this literature often finds discrepancies between such measurements [38]. However, the literature exploring the validity or reliability of travel diaries is much smaller. Panter et al. have explored this issue in a recent study and their work suggested diaries led to overestimation of active commuting [40]. We have no reason to think the travel diary data from the NTS are abnormally unreliable or lack external validity, nor is there reason to believe that their quality would vary by type of physical environment the respondent lived in. However, we acknowledge that the data are based on self-report which may be subject to bias and inaccuracy.

Our study used cross-sectional data, which does not allow the inference of causality. Natural experiments have been shown to be better suited to establish determinants, rather than correlates, of physical activity [41]. However, natural experiments are designed to study particular behaviours (e.g. participation in active travel) before and after plausibly-related exogenous changes (e.g. new traffic infrastructure), and the results are therefore frequently specific to a particular locality and not necessarily transferrable to a broader population. In this paper we were interested in inequalities in active travel at the population-level. In terms of assessing health-relevant longitudinal changes in the physical environment it would be particularly challenging to collect comparable periodical data on a variety of measures such as captured by our indicator of environmental deprivation [27].

There have been attempts in the UK to apply a health framework to transport policies, with the aim of integrating perspectives related to economy, regeneration, social justice and health. Yet, UK transport policy has largely focused on improving conditions for motorised transport, neglecting interests of those participating in active travel, particularly in areas characterised by economic decline and social exclusion [42]. Changing broader social and environmental conditions for the whole of society might result in creating time, space and capacities for individuals to reconsider the feasibility of active transport. It might also mitigate the socially patterned risks involved for those who do actively travel [42].

## Conclusions

This research found that the likelihood of choosing an active travel mode for short trips was relatively high for low income people in the most environmentally-disadvantaged areas, and relatively low for high income people in the least environmentally deprived areas. This suggests that physical environment, as measured in this work, is not a strong influence on socio-economic inequalities in active travel. Nevertheless improvements in the physical environment may mitigate the risks for those who actively travel and continue to encourage such forms of transport in the face of increasing car ownership.
